# Hibernating ribosomes as drug targets?

**DOI:** 10.3389/fmicb.2024.1436579

**Published:** 2024-07-29

**Authors:** Chinenye L. Ekemezie, Sergey V. Melnikov

**Affiliations:** ^1^Biosciences Institute, Newcastle University, Newcastle upon Tyne, United Kingdom; ^2^Medical School of Newcastle University, Newcastle upon Tyne, United Kingdom

**Keywords:** hibernation, ribosome, ribosome-targeting drugs, dormancy, antimicrobial resistance

## Abstract

When ribosome-targeting antibiotics attack actively growing bacteria, they occupy ribosomal active centers, causing the ribosomes to stall or make errors that either halt cellular growth or cause bacterial death. However, emerging research indicates that bacterial ribosomes spend a considerable amount of time in an inactive state known as ribosome hibernation, in which they dissociate from their substrates and bind to specialized proteins called ribosome hibernation factors. Since 60% of microbial biomass exists in a dormant state at any given time, these hibernation factors are likely the most common partners of ribosomes in bacterial cells. Furthermore, some hibernation factors occupy ribosomal drug-binding sites – leading to the question of how ribosome hibernation influences antibiotic efficacy, and vice versa. In this review, we summarize the current state of knowledge on physical and functional interactions between hibernation factors and ribosome-targeting antibiotics and explore the possibility of using antibiotics to target not only active but also hibernating ribosomes. Because ribosome hibernation empowers bacteria to withstand harsh conditions such as starvation, stress, and host immunity, this line of research holds promise for medicine, agriculture, and biotechnology: by learning to regulate ribosome hibernation, we could enhance our capacity to manage the survival of microorganisms in dormancy.

## Ribosomes, common drug targets, are likely to exist primarily in a dormant state

Ribosomes are complex molecular machines that are abundant and essential in all cells due to their requirement for protein synthesis. It is, therefore, not surprising that bacterial ribosomes serve as a major target for antibacterial drugs. In the United Kingdom, ribosome-targeting drugs account for 25% of antimicrobial prescriptions by general medical practices, and on a global scale, they make up about 60% of the currently approved antimicrobial (Dolk et al., 2018; Lin et al., 2018).

**Figure 1:**
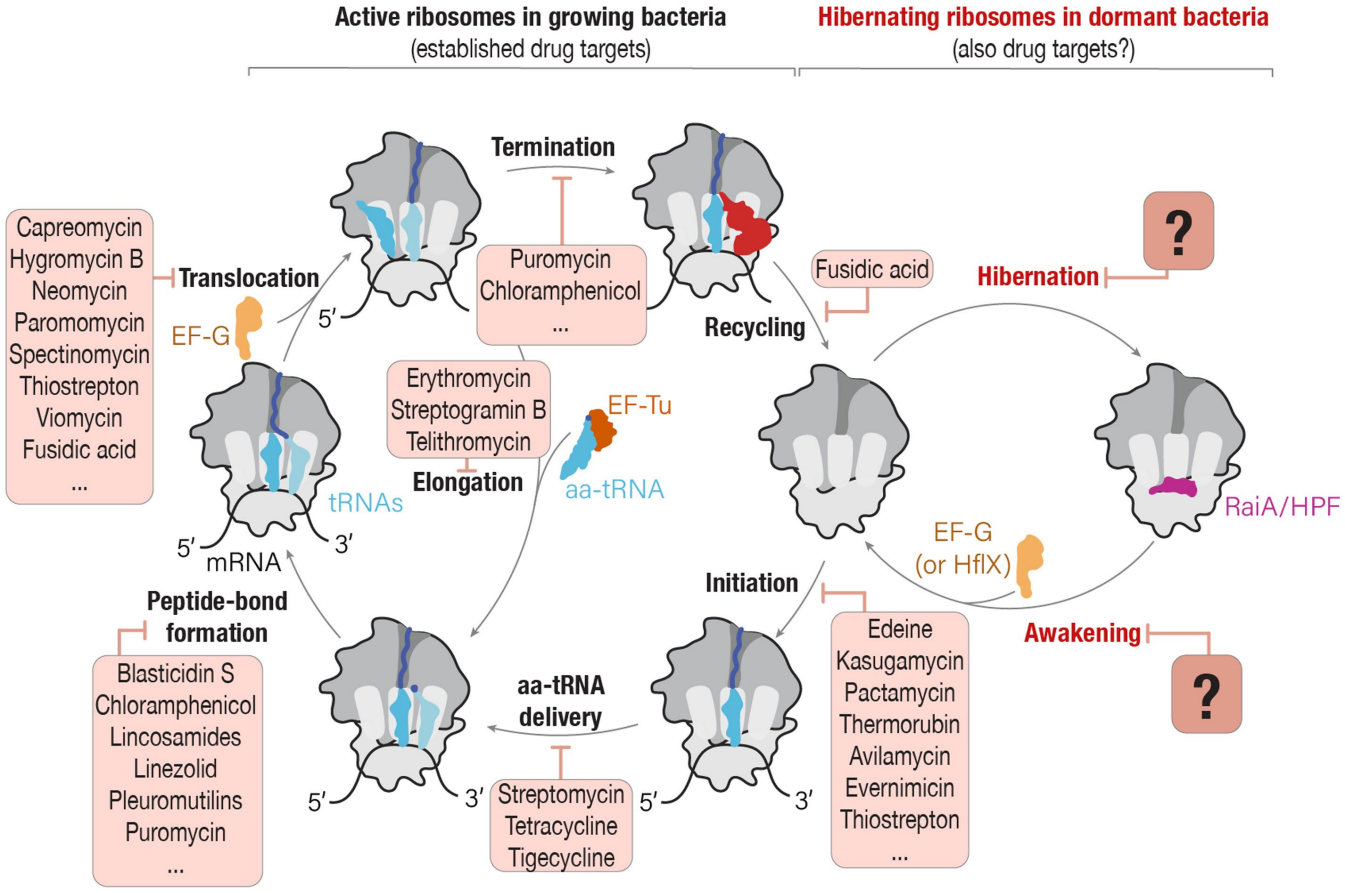
Can drugs that target active ribosomes in growing cells also target hibernating ribosomes in dormant cells? The schematic illustrates two phases of ribosome activity: active protein synthesis and ribosome hibernation. In actively growing bacteria, ribosomes participate in protein synthesis, and each step of this process—from the initiation of protein synthesis until ribosome recycling—is a known target of ribosome-targeting antibiotics. However, during episodes of cellular stress or nutrient deprivation, ribosomes enter a hibernation state in which they associate with hibernation factors. Despite ribosome hibernation being a widespread phenomenon that is crucial for the ability of pathogenic organisms to endure hostile environments, we know little about the potential ability of ribosome-targeting drugs to interfere with the ribosome’s entry or exit from hibernation. Source: Adapted from “A molecular network of conserved factors keeps ribosomes dormant in the egg” by Leesch et al. (2023) https://doi.org/10.1038/s41586-022-05623-y, with permission from Springer Nature under license no. 5847220809267.

Currently, our understanding of the mechanisms by which ribosome-targeting drugs exert their inhibitory or toxic activities relies primarily on the studies of active bacterial ribosomes (Wilson, 2014; Lin et al., 2018). These studies showed that most families of ribosome-targeting drugs bind to the very few sites of the ribosome where they overlap with the normal position of ribosomal ligands, including mRNA, tRNA, or the nascent peptide produced by the ribosome (Lin et al., 2018; Figure 1). Consequently, ribosome-targeting drugs were shown to prevent ribosome from binding or dissociation from its ligands, thereby causing an arrested or inaccurate protein synthesis and leading to growth inhibition or death of bacterial cells (Wilson, 2014).

However, this understanding of ribosome targeting with antimicrobials tends to overlook the fact that ribosomes do not remain constitutively active. When cells face starvation, stress, or other unfavorable conditions, ribosomes disengage from their substrates, such as mRNAs and tRNAs, and bind to a specialized class of proteins known as ribosome hibernation factors (Vila-Sanjurjo et al., 2004; Figure 1). These hibernation factors prevent ribosomes from undesired activity (as was observed in animal oocytes) or degradation by nucleases (as was observed in several bacterial species), thus enabling dormant cells to rapidly reawaken when conditions improve (Feaga et al., 2020; Feaga and Dworkin, 2021; Lipońska and Yap, 2021; Prossliner et al., 2021; Leesch et al., 2023). And because more than 60% of the Earth’s microbial biomass is estimated to exist in various forms of dormancy, it is likely that ribosome hibernation is the predominant state of bacterial ribosomes in nature (Blagodatskaya and Kuzyakov, 2013; Rittershaus et al., 2013).

Although it is currently unclear whether ribosome hibernation factors can compete with drugs for ribosome binding (Li et al., 2021), these factors have been identified in every organism tested so far. In the most studied bacteria, *Escherichia coli*, at least seven proteins were shown to serve as hibernation factors, including proteins RMF, HPF, RaiA, Sra, YqjD, ElaB and YgaM (Wada et al., 1990, 1995; Agafonov et al., 1999, 2001; Yoshida et al., 2012). Furthermore, proteins HPF and RaiA (that belong to the same protein family, RaiA/HPF) were found in virtually all bacterial species (Helena-Bueno et al., 2024a,b). In other bacteria, including pathogenic Mycobacteria, ribosomes were shown to associate with an additional hibernation factor, Balon (Helena-Bueno et al., 2024b). Aside from bacteria, hibernation factors have been identified in metabolically inactive eukaryotic cells, including stressed or stationary cell cultures, as well as fungal spores and animal embryos. These families of ribosome hibernation factors include Stm1/SERPB1 (Ben-Shem et al., 2011; Anger et al., 2013), Lso2/CCDC124 (Wang et al., 2018; Ehrenbolger et al., 2020; Wells et al., 2020), IFRD1/IFRD2 (Brown et al., 2018; Hopes et al., 2022), MDF1 (Barandun et al., 2019; Nicholson et al., 2022; McLaren et al., 2023), MDF2 (Barandun et al., 2019), and Dapl1 (Leesch et al., 2023), pointing to a great structural diversity and potentially a much higher number of currently unknown hibernation factors in nature.

Thus, despite the fact that most ribosomes exist in a dormant state, almost all studies detailing antibiotic mechanisms of action focus on active ribosomes without hibernation factors present. Likewise, studies of ribosome hibernation have largely been conducted in the absence of antibiotics. It is clear that these two classes of ribosome-binding entities have a large potential to affect each other, though our understanding of these interactions is still in its infancy. In this review, we discuss the current state of knowledge on the interactions between ribosome hibernation and antibiotic efficacy, and explore hibernating ribosomes as a potential untapped drug target.

## Ribosome-targeting antibiotics occupy the binding sites of certain hibernation factors

Most ribosome-targeting antibiotics bind to ribosomal active centers (Wilson, 2009; Wilson, 2014; Lin et al., 2018; Beckert et al., 2021; Paternoga et al., 2023), generally blocking ribosomes from properly associating with or dissociating from their substrates. Likewise, each of the three well-characterized bacterial ribosome hibernation factor protein families has been shown to occupy ribosomal active centers, including tRNA-binding sites and the mRNA channel (Helena-Bueno et al., 2024a). These parallel observations raise the question of whether ribosome-targeting drugs can block hibernation factors from binding ribosomes, or vice versa – and how these interactions might affect the ability of antibiotic-targeted pathogens to survive states of dormancy.

**Figure 2:**
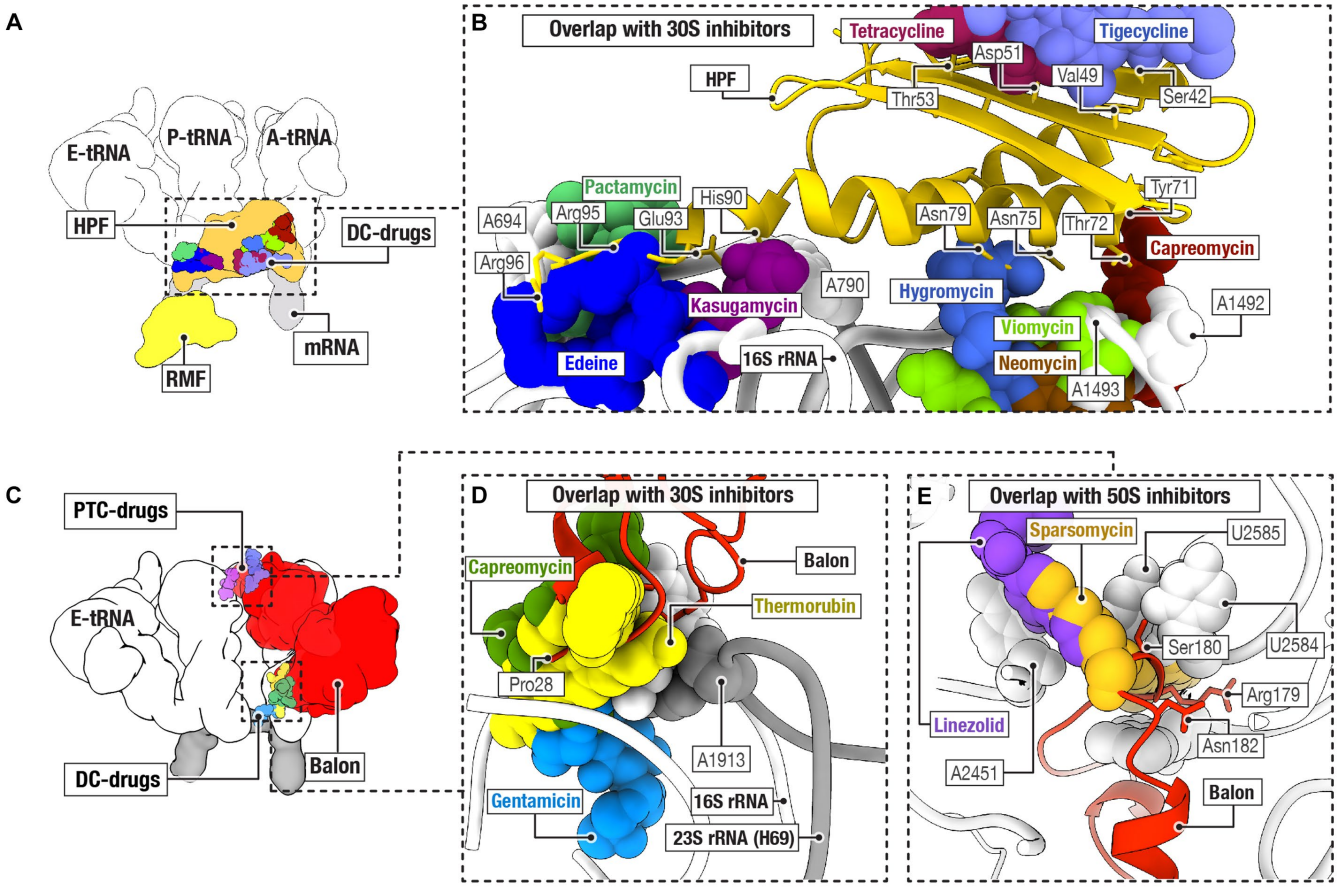
Ribosome hibernation factors can occupy ribosomal drug-binding sites. **(A)** Schematic illustration compares the ribosomal binding sites for the hibernation factors RMF and HPF, and for tRNAs (labeled as E, P, and A), mRNA, and ribosome-targeting antibiotics that bind to the decoding center (DC-drugs) and the ribosomal mRNA channel. **(B)** Close-up view illustrating the overlap between the ribosomal binding sites for hibernation factor HPF and several families of ribosome-targeting antibiotics. **(C)** Schematic illustration of the location of the binding site of Balon relative to the binding sites of ribosome-targeting antibiotics (labeled as PTC-drugs and DC-drugs to indicate their binding to the peptidyl-transferase center and the decoding center of the ribosome, respectively). **(D,E)** Close-up views illustrating the overlap between the ribosomal binding sites for hibernation factor Balon and several families of ribosome-targeting antibiotics in the small ribosomal subunit **(D)** and the large ribosomal subunit **(E)**.

In bacteria, three protein families of hibernation factors have been structurally characterized so far as a complex with ribosomes. These include protein families RaiA/HPF, RMF, and Balon (Figure 2). Comparative structural analyses have revealed that two of the three families of bacterial ribosome hibernation factors—the universally conserved RaiA/HPF and widely occurring Balon—substantially overlap with the binding positions of multiple families of ribosome-targeting antibiotics (Figures 2A–E). In particular, the hibernation factor RaiA (also known as protein Y and YfiA) occupies the mRNA channel as well as the binding sites for the A- and P-tRNAs in the small ribosomal subunit. There, RaiA/HPF overlaps with an array of ribosome-targeting antibiotics, including neomycin (Borovinskaya et al., 2007), gentamicin (Borovinskaya et al., 2007), hygromycin B (Borovinskaya et al., 2008), capreomycin (Stanley et al., 2010), tetracycline (Brodersen et al., 2000), and tigecycline (Jenner et al., 2013), kasugamycin (Schuwirth et al., 2006), pactamycin (Dinos et al., 2004), and edeine (Dinos et al., 2004; Figures 2A,B). Similarly, the hibernation factor Balon binds the ribosomal A site by contacting both the decoding center of the small subunit and the peptidyl transfer center of the large subunit (Helena-Bueno et al., 2024b). There, Balon occupies the binding sites for such antibiotics as thermorubin (Bulkley et al., 2012), hygromycin B (Borovinskaya et al., 2008), amikacin (Seely et al., 2023), neomycin (Borovinskaya et al., 2007), gentamicin (Borovinskaya et al., 2007), paromomycin (Carter et al., 2000), capreomycin (Stanley et al., 2010), and viomycin (Stanley et al., 2010) in the small ribosomal subunit, as well as blasticidin (Svidritskiy et al., 2013), linezolid (Wilson et al., 2008), chloramphenicol (Schlünzen et al., 2001), clindamycin (Dunkle et al., 2010), dalfopristin (Noeske et al., 2014), avilamycin (Arenz et al., 2016), evernimycin (Arenz et al., 2016), and thiostrepton (Walter et al., 2012) in the large ribosomal subunit (Figures 2C–E).

In contrast, the hibernation factor RMF, which is found in proteobacteria, does not overlap with any of the ribosome-targeting drug binding sites identified so far (Polikanov et al., 2012; Figures 2A,B). RMF binds to the ribosome in the mRNA channel of the small subunit, in the vicinity of the mRNA-binding anti-Shine-Dalgarno sequence in the 16S rRNA. This places RMF more than 10 Å away from the closest antibiotic-binding site for the drug edeine (Schuwirth et al., 2006; Figures 2A,B). There are also several families of ribosome-targeting antibiotics that bind at a substantial distance from every characterized binding site for hibernation factors. In the large ribosomal subunit, these include drugs that target the nascent peptide tunnel of the ribosome, including macrolides(Tu et al., 2005), ketolides (Tu et al., 2005), streptogramin B (Tu et al., 2005), and tetracenomycin (Osterman et al., 2020). These also include certain drugs that target the ribosomal peptidyl-transferase center: retapamulin (Davidovich et al., 2007), tiamulin (Davidovich et al., 2007), and clindamycin (Dunkle et al., 2010). Similarly, in the small subunit, spectinomycin (Borovinskaya et al., 2007) binds away from the hibernation factors. Therefore, while there are several examples of direct physical overlap, in many cases hibernation factors and ribosome-targeting antibiotics seem physically capable of binding ribosomes simultaneously.

In addition to hibernation factors, the process of ribosome hibernation can involve additional ribosome-binding proteins, including the elongation factors EF-Tu, EF-G in bacteria and eEF2 in eukaryotes (Anger et al., 2013; Feaga et al., 2020; Smith et al., 2022; Helena-Bueno et al., 2024b). Proteins EF-Tu and EF-G are targets for such antibiotics as elfamycins (Prezioso et al., 2017) and fusidic acid (Laurberg et al., 2000), respectively, suggesting a potential interplay between these antibiotics and the process of ribosome hibernation that has yet to be experimentally elucidated.

These structural analyses raise the question: what happens when ribosome targeting drugs and hibernation factors are both available for ribosome binding? What is the relative affinity of antibiotics and hibernation factors to the ribosome? Can ribosome hibernation factors protect ribosomes from binding to antibiotics? Conversely, can antibiotics prevent ribosomes from entering or exiting the state of hibernation, thereby causing ribosome instability or irreversible hibernation in dormant bacterial cells?

## Hibernation factors may protect dormant bacteria from certain ribosome-targeting drugs

Given their role in protecting a central molecular machine required for cellular survival and growth, perhaps it is not surprising that ribosome hibernation factors empower pathogenic bacteria to endure hostile environments, including stress, starvation, and assaults with antibiotics (Trauner et al., 2012; Lipońska and Yap, 2021; Prossliner et al., 2021). For example, in *Mycobacterium smegmatis*, inactivation of ribosome hibernation mechanisms accelerates ribosome degradation and provokes bacterial intolerance to hypoxia. This finding is especially remarkable because hypoxic and dormant *Mycobacteria* frequently cause latent infections in human tissues, consistently posing a threat of active tuberculosis disease in up to two billion people worldwide (Lin and Flynn, 2010).

**Figure 3:**
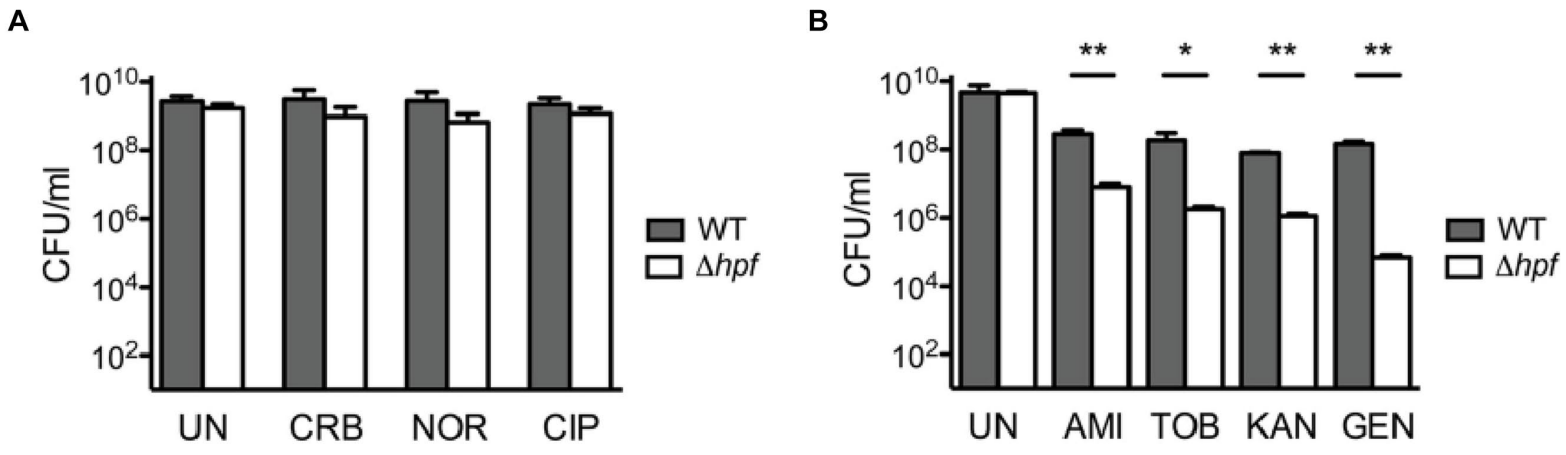
Loss of hibernation factors in dormant bacteria potentiates aminoglycoside-mediated toxicity. **(A)** When cultures of wild-type (WT) and HPF-deficient *Listeria monocytogenes* are cultured for 72 h in stationary phase and then treated with various antibiotics, they show similar tolerance to the non-ribosome-targeting drugs carbenicillin (CRB), norfloxacin (NOR), and ciprofloxacin (CIP). UN indicates untreated cultures, and CFU stands for colony-forming units. **(B)** However, when stationary *L. monocytogenes* cultures are treated with aminoglycoside antibiotics, the HPF-deficient strain shows up to 3 orders of magnitude reduction in CFU compared to WT. Labels indicate the aminoglycoside antibiotics amikacin (AMI), tobramycin (TOB), kanamycin (KAN), and gentamicin (GEN) [this figure is reproduced from Ref (McKay and Portnoy, 2015) with permission from the American Association for Microbiology, license ID 1474012-1].

It is currently unclear whether hibernation factors can directly compete with antibiotics for ribosome binding. However, it has been established that hibernation factors can protect bacteria from the toxicity of aminoglycoside antibiotics that target the ribosome. In one study, stationary phase *Listeria monocytogenes* with a knockout of the hibernation factor HPF were markedly more susceptible to aminoglycosides (either tobramycin, gentamicin, amikacin, or neomycin), but not non-ribosomal antibiotics (such as ciprofloxacin, carbenicillin, and norfloxacin), compared to the wild-type (WT) strain (McKay and Portnoy, 2015). Interestingly, this phenotype was neutralized when aminoglycosides were used in combination with a protein synthesis inhibitor (chloramphenicol) and an RNA synthesis inhibitor (rifampicin). The mechanism of this phenomenon is currently unclear. One possible explanation is that residual ribosomal activity in the absence of hibernation factors makes bacteria vulnerable to aminoglycosides – likely because these antibiotics cause cell death through errors in protein synthesis (McKay and Portnoy, 2015). However, an equally valid explanation is that loss of HPF results in lower ribosome abundance, which would make the bacteria more susceptible to translation inhibition (Figure 3).

Similarly, *M. smegmatis* lacking HPF (also known as mpY or MSMEG_1878) exhibited a 100-fold reduction in colony-forming ability after either streptomycin or kanamycin treatment under zinc starvation conditions (Li et al., 2018). Meanwhile, overexpression of RaiA in *V. cholerae* increased tolerance to the aminoglycoside antibiotics tobramycin, gentamicin, and neomycin, but decreased sensitivity to non-ribosomal inhibitors, such as trimethoprim, ciprofloxacin, or carbenicillin (Lang et al., 2021). Together, these findings reveal a common pattern whereby depletion of hibernation factors increases bacterial sensitivity to aminoglycosides but not to other antibiotics that target other cellular components.

While we are only beginning to explore the potential interplay between ribosome hibernation factors and antibiotic sensitivity, these compelling examples illustrate how hibernation factors can dramatically increase the ability of dormant bacteria to withstand antibiotic toxicity – in particular for ribosome-targeting antibiotics like aminoglycosides in dormant bacteria. Whether a direct competition exists between ribosome hibernation factors and the ribosome targeting antibiotics would only be clear through direct analyses of this potential competition using biochemistry or other experimental techniques.

## Can resistance-conferring mutations in rRNA impact hibernation factors binding?

Because some rRNA residues bind not only antibiotics but also ribosome hibernation factors, the question arises: can mutations that confer resistance to ribosome-targeting drugs also impact ribosome affinity for hibernation factors? While there is no experimental evidence to answer this question, some insights can be obtained from the literature. Previously, ribosome hibernation factors Balon and HPF were shown to bind 87 rRNA residues (Helena-Bueno et al., 2024b). Among these rRNA residues, 13 were shown to bind ribosome-targeting antibiotics and bear mutations in drug-resistant bacteria (Table 1). Seven of these residues bind to Balon and include C1409 and G1491 in the 16S rRNA, and A1067, A1095, A2451, C2452, and A2469 in the 23S rRNA. The remaining six residues bind to RaiA/HPF and include C795, U965, G966, U1052, U1495, and C1496 in the 16S rRNA (Table 1). Mutations of each of these residues result in lower ribosome affinity for antibiotics and can lead to 500 times higher minimum inhibitory concentrations (Table 1). It, therefore, seems possible that some of these mutations may also impact ribosome affinity for hibernation factors. If correct, this hypothesis would mean that ribosomes in some drug-resistant bacteria may have a lower affinity to hibernation factors and, therefore, a higher propensity for degradation by nucleases leading to lower tolerance to dormancy.

**Table 1:** Hibernation factors bind some rRNA residues that mutate in drug-resistant ribosomes.

HPF residues that interact with rRNA residues whose mutations confer bacterial drug resistance					
HPF residue	rRNA contact	Drugs that bind to the rRNA contact	Model organism	Resistance conferring mutations (compared to wild-type strain)	References
Arg102	C795 (16S)	Ede, Kas, Pac	*Halobacterium halobium*	C795U required 80 times more pactamycin (80 μM) to cause lethality.	Mankin (1997)
His8, Ile40, Glu59	U965 (16S)	Tet, Tig	*Helicobacter pylori*	A965G produced 100 times more colonies in the presence of 2 μg/mL of tetracycline.	Dailidiene et al. (2002)
Ile40, Lys57, Glu59, Ile61, Phe70	G966 (16S)	Tet, Tig	*Escherichia coli*	G966U required 4 times more tetracycline or tigecycline to arrest cell growth.	Bauer et al. (2004) and Polikanov et al. (2014)
Ser46, Ala48	U1052 (16S)	Tet, Tig, Neg	*Escherichia coli*	U1052G required approximately 4.5 times more negamycin to kill 50% of the population.	Cocozaki et al. (2016)
Lys26	U1495 (16S)	Ami, Gen, Hyg B, Neo, Vio	*Mycobacterium smegmatis*	U1495C and U1495A required 512 times more paromomycin, and lividomycin to arrest cell growth.	Hobbie et al. (2006)
Lys26	C1496 (16S)	Ami, Gen, Hyg B, Neo, Vio	*Mycobacterium smegmatis*	C1496U required 32 times more hygromycin B to arrest cell growth.	Pfister et al. (2003)

Balon residues that interact with rRNA residues whose mutations confer bacterial drug resistance					
Balon residue	rRNA contact	Drugs that bind to the rRNA contact	Model organism	Resistance conferring mutation (compared to wild-type strain)	References
Glu26	C1409 (16S)	Ami, Cap, Gen, Par, The	*Thermus thermophilus*	C1409G required 200 times more kanamycin to arrest cell growth.	Pfister et al. (2005)
			*Mycobacterium smegmatis*	C1409U required at least 16 times more neomycin, gentamicin, tobramycin and kanamycin to arrest cell growth.	Gregory et al. (2005)
	G1491 (16S)	Neo, Par, Cap	*Mycobacterium smegmatis*	G1491C required at least 512 times more paromomycin and lividomycin to arrest cell growth.	Hobbie et al. (2006)
His27, Pro28			*Mycobacterium smegmatis*	G1491A required 64 times more paromomycin to arrest cell growth.	Kalapala et al. (2010)
			*Mycobacterium smegmatis*	G1491U required at least 512 times more paromomycin and geneticin to arrest cell growth.	Pfister et al. (2005)
Gly296, Asn344, Asn345, Arg368	A1067 (23S)	Thi	*Escherichia coli*	Ribosomes bearing A1067C and A1067U have ~65% lower thiostrepton affinity.	Thompson et al. (1988)
Arg368, Tyr369	A1095 (23S)	Thi	*Escherichia coli*	Ribosomes bearing A1095U or A1095C have significantly lower affinity for thiostrepton.	Xu et al. (2002)
Gly176, Ser177, Asp178	A2451 (23S)	Car, Chl, Cli, Dal, Pur, Spa, Tia, Vir	*Thermus thermophilus*	In a disc diffusion assay, A2451U showed no zone of inhibition to tiamulin and chloramphenicol.	Killeavy et al. (2020)
Gly176, Ser177	C2452 (23S)	Car, Chl, Cli, Dal, Pur, Spa, Tia, Vir	*Thermus thermophilus*	In a disc diffusion assay, C2452U showed no zone of inhibition to tiamulin.	Killeavy et al. (2020)
Lys207	A2469 (23S)	Avi, Eve	*Streptococcus pneumoniae*	A2469C required at least 16 times more avilamycin to arrest cell growth.	Adrian et al. (2000)

Here, we list 13 rRNA residues that have the following two characteristics: (i) They participate in binding to both hibernation factors and antibiotics. (ii) And they undergo resistance-conferring mutations in clinical isolates or laboratory engineered bacterial strains. The abbreviations for the antibiotic names in the table are as follows: Ami, amikacin; Avi, avilamycin; Car, carbomycin; Cap, capreomycin; Chl, chloramphenicol; Cli, clindamycin; Dal, dalfopristin; Ede, edeine; Eve, evernimycin; Gen, gentamicin; Hyg, hygromycin B; Kas, kasugamycin; Neg, negamycin; Neo, neomycin; Pac, pactamycin; Par, paromomycin; Pur, puromycin; Spa, sparsomycin; Tet, tetracycline; The, thermorubin; Thi, Thiostrepton; Tia, tiamulin; Tig, tigecycline; Vir, virginiamycin; Vio, viomycin.

## Future outlooks and outstanding questions

Because ribosome hibernation factors are crucial for ribosome stability in dormant bacteria, it is logical to hypothesize that preventing these factors from binding to ribosomes should cause slow but irreparable ribosome degradation in dormant cells. This seems to be especially relevant to human pathogens that rely on a long-term dormancy for their survival and infectivity, which include *Mycobacterium tuberculosis*, *Clostridioides difficile*, *Salmonella* species, as well as the pathogenic eukaryotes *Giardia lamblia*, among others (Dworkin and Shah, 2010). Furthermore, beyond infectious disease, if this hypothesis is correct, it would be relevant to many other living systems, such as plant seeds that remain fertile for more than four seasons (Trusiak et al., 2022), or human embryos that can stay dormant for over three decades (Kessel, 1966; Burkholder et al., 1971). In these cells and organisms, ribosome hibernation factors—such as Serpb1 and Dapl1 in eukaryotes and RMF and RaiA/HPF in bacteria—prevent ribosome degradation and ensure the viability of dormant cells (Prossliner et al., 2021; Leesch et al., 2023).

The fact that the genetic knockouts of ribosome hibernation factors were shown to dramatically impair the viability of organisms after a few days of dormancy, leads to the following question: can we achieve the same phenotype of the absence of ribosome hibernation factors by using small molecules that prevent the binding of hibernation factors to ribosomes?

Currently, it is widely accepted that dormant pathogens are highly tolerant to drugs (Boeck, 2023). However, the current tests of antibiotic toxicity typically involve relatively short-term exposures of cells to drugs, which range from 30 min to a few hours of drug administration (McKay and Portnoy, 2015; Li et al., 2018; Song and Wood, 2020; Lang et al., 2021). As a result, we know little about how dormant cells react to long-term treatment with drugs, including ribosome-targeting antibiotics. This is particularly important because genetic knockouts of ribosome hibernation factors show that their loss has only a minor impact on the stability of ribosomes and the viability of bacteria after short-term stress. Specifically, in dormant *E. coli*, it takes at least several days for most rRNAs to degrade in the absence of the hibernation factors RMF and RaiA/HPF (Prossliner et al., 2021). Therefore, it would be logical to expect that—if ribosome-targeting drugs can indeed displace ribosome hibernation factors in dormant bacteria—the measurable impact of these drugs on ribosome stability and the viability of dormant cells would require to several days, rather than several minutes of hours, of continuous drug administration.

To test whether hibernating ribosomes can indeed serve as drug targets we will need to answer the following questions:

- How does the affinity of antibiotics and hibernation factors to the ribosome compare to each other?
- Can ribosome-targeting antibiotics displace ribosome hibernation factors from the ribosome in dormant cells and organisms?
- If so, what concentrations are required for this displacement and how do they compare with the minimum inhibitory concentrations for these ribosome-targeting drugs?
- If one or a combination of ribosome-targeting drugs can indeed displace hibernation factors from the ribosome *in dormant cells*, does it lead to ribosome instability?
- And if so, how long does it take for the ribosome to degrade and thereby impair the recovery of cells from dormancy?
- And finally, because the translation factors EF-Tu and eEF2 also participate in ribosome hibernation, can their targeting with their corresponding drugs—such as elfamycins and fusidic acid, respectively—prevent ribosomes from entering or exiting hibernation?

This line of research is risky, but it seems worthy further exploring given the ubiquitous presence of hibernation states of cells and organisms in nature and the potential impact of successful targeting of ribosome dormancy on medicine, agriculture and biotechnology.

## Author contributions

SM: Writing – original draft, Writing – review & editing. CE: Writing – original draft, Writing – review & editing.

## References

[ref1] Adrian P. V. Mendrick C. Loebenberg D. McNicholas P. Shaw K. J. Klugman K. P.. (2000). Evernimicin (SCH27899) inhibits a novel ribosome target site: analysis of 23S ribosomal DNA mutants. Antimicrob. Agents Chemother. 44, 3101–3106. doi: 10.1128/AAC.44.11.3101-3106.2000, PMID: 11036030 PMC101610

[ref2] Agafonov D. E. Kolb V. A. Nazimov I. V. Spirin A. S. (1999). A protein residing at the subunit interface of the bacterial ribosome. Proc. Natl. Acad. Sci. 96, 12345–12349. doi: 10.1073/pnas.96.22.12345, PMID: 10535924 PMC22919

[ref3] Agafonov D. E. Kolb V. A. Spirin A. S. (2001). Ribosome-associated protein that inhibits translation at the aminoacyl-tRNA binding stage. EMBO Rep. 2, 399–402. doi: 10.1093/embo-reports/kve091, PMID: 11375931 PMC1083885

[ref4] Anger A. M. Armache J.-P. Berninghausen O. Habeck M. Subklewe M. Wilson D. N.. (2013). Structures of the human and Drosophila 80S ribosome. Nature 497, 80–85. doi: 10.1038/nature12104 23636399

[ref5] Arenz S. Juette M. F. Graf M. Nguyen F. Huter P. Polikanov Y. S.. (2016). Structures of the orthosomycin antibiotics avilamycin and evernimicin in complex with the bacterial 70S ribosome. Proc. Natl. Acad. Sci. 113, 7527–7532. doi: 10.1073/pnas.1604790113 27330110 PMC4941455

[ref6] Barandun J. Hunziker M. Vossbrinck C. R. Klinge S. (2019). Evolutionary compaction and adaptation visualized by the structure of the dormant microsporidian ribosome. Nat. Microbiol. 4, 1798–1804. doi: 10.1038/s41564-019-0514-6, PMID: 31332387 PMC6814508

[ref7] Bauer G. Berens C. Projan S. J. Hillen W. (2004). Comparison of tetracycline and tigecycline binding to ribosomes mapped by dimethylsulphate and drug-directed Fe2+ cleavage of 16S rRNA. J. Antimicrob. Chemother. 53, 592–599. doi: 10.1093/jac/dkh125, PMID: 14985271

[ref8] Beckert B. Leroy E. C. Sothiselvam S. Bock L. V. Svetlov M. S. Graf M.. (2021). Structural and mechanistic basis for translation inhibition by macrolide and ketolide antibiotics. Nat. Commun. 12:4466. doi: 10.1038/s41467-021-24674-9, PMID: 34294725 PMC8298421

[ref9] Ben-Shem A. Garreau de Loubresse N. Melnikov S. Jenner L. Yusupova G. Yusupov M. (2011). The structure of the eukaryotic ribosome at 3.0 Å resolution. Science 334, 1524–1529. doi: 10.1126/science.1212642, PMID: 22096102

[ref10] Blagodatskaya E. Kuzyakov Y. (2013). Active microorganisms in soil: critical review of estimation criteria and approaches. Soil Biol. Biochem. 67, 192–211. doi: 10.1016/j.soilbio.2013.08.024

[ref11] Boeck L. (2023). Antibiotic tolerance: targeting bacterial survival. Curr. Opin. Microbiol. 74:102328. doi: 10.1016/j.mib.2023.102328 37245488

[ref12] Borovinskaya M. A. Pai R. D. Zhang W. Schuwirth B. S. Holton J. M. Hirokawa G.. (2007). Structural basis for aminoglycoside inhibition of bacterial ribosome recycling. Nat. Struct. Mol. Biol. 14, 727–732. doi: 10.1038/nsmb1271 17660832

[ref13] Borovinskaya M. A. Shoji S. Fredrick K. Cate J. H. (2008). Structural basis for hygromycin B inhibition of protein biosynthesis. RNA 14, 1590–1599. doi: 10.1261/rna.1076908, PMID: 18567815 PMC2491480

[ref14] Brodersen D. E. Clemons W. M. Carter A. P. Morgan-Warren R. J. Wimberly B. T. Ramakrishnan V. (2000). The structural basis for the action of the antibiotics tetracycline, pactamycin, and hygromycin B on the 30S ribosomal subunit. Cell 103, 1143–1154. doi: 10.1016/S0092-8674(00)00216-6, PMID: 11163189

[ref15] Brown A. Baird M. R. Yip M. C. Murray J. Shao S. (2018). Structures of translationally inactive mammalian ribosomes. eLife 7:e40486. doi: 10.7554/eLife.40486 30355441 PMC6226290

[ref16] Bulkley D. Johnson F. Steitz T. A. (2012). The antibiotic thermorubin inhibits protein synthesis by binding to inter-subunit bridge B2a of the ribosome. J. Mol. Biol. 416, 571–578. doi: 10.1016/j.jmb.2011.12.055, PMID: 22240456 PMC3336878

[ref17] Burkholder G. D. Comings D. E. Okada T. A. (1971). A storage form of ribosomes in mouse oocytes. Exp. Cell Res. 69, 361–371. doi: 10.1016/0014-4827(71)90236-9, PMID: 4131262

[ref18] Carter A. P. Clemons W. M. Brodersen D. E. Morgan-Warren R. J. Wimberly B. T. Ramakrishnan V. (2000). Functional insights from the structure of the 30S ribosomal subunit and its interactions with antibiotics. Nature 407, 340–348. doi: 10.1038/35030019 11014183

[ref19] Cocozaki A. I. Altman R. B. Huang J. Buurman E. T. Kazmirski S. L. Doig P.. (2016). Resistance mutations generate divergent antibiotic susceptibility profiles against translation inhibitors. Proc. Natl. Acad. Sci. 113, 8188–8193. doi: 10.1073/pnas.1605127113, PMID: 27382179 PMC4961145

[ref20] Dailidiene D. Bertoli M. T. Miciuleviciene J. Mukhopadhyay A. K. Dailide G. Pascasio M. A.. (2002). Emergence of tetracycline resistance in *Helicobacter pylori*: multiple mutational changes in 16S ribosomal DNA and other genetic loci. Antimicrob. Agents Chemother. 46, 3940–3946. doi: 10.1128/AAC.46.12.3940-3946.2002, PMID: 12435699 PMC132778

[ref21] Davidovich C. Bashan A. Auerbach-Nevo T. Yaggie R. D. Gontarek R. R. Yonath A. (2007). Induced-fit tightens pleuromutilins binding to ribosomes and remote interactions enable their selectivity. Proc. Natl. Acad. Sci. 104, 4291–4296. doi: 10.1073/pnas.0700041104, PMID: 17360517 PMC1817833

[ref22] Dinos G. Wilson D. N. Teraoka Y. Szaflarski W. Fucini P. Kalpaxis D.. (2004). Dissecting the ribosomal inhibition mechanisms of edeine and pactamycin: the universally conserved residues G693 and C795 regulate P-site RNA binding. Mol. Cell 13, 113–124. doi: 10.1016/S1097-2765(04)00002-4, PMID: 14731399

[ref23] Dolk F. C. K. Pouwels K. B. Smith D. R. Robotham J. V. Smieszek T. (2018). Antibiotics in primary care in England: which antibiotics are prescribed and for which conditions? J. Antimicrob. Chemother. 73:ii2–ii10. doi: 10.1093/jac/dkx504, PMID: 29490062 PMC5890730

[ref24] Dunkle J. A. Xiong L. Mankin A. S. Cate J. H. (2010). Structures of the *Escherichia coli* ribosome with antibiotics bound near the peptidyl transferase center explain spectra of drug action. Proc. Natl. Acad. Sci. 107, 17152–17157. doi: 10.1073/pnas.1007988107, PMID: 20876128 PMC2951456

[ref25] Dworkin J. Shah I. M. (2010). Exit from dormancy in microbial organisms. Nat. Rev. Microbiol. 8, 890–896. doi: 10.1038/nrmicro2453, PMID: 20972452

[ref26] Ehrenbolger K. Jespersen N. Sharma H. Sokolova Y. Y. Tokarev Y. S. Vossbrinck C. R.. (2020). Differences in structure and hibernation mechanism highlight diversification of the microsporidian ribosome. PLoS Biol. 18:e3000958. doi: 10.1371/journal.pbio.3000958, PMID: 33125369 PMC7644102

[ref27] Feaga H. A. Dworkin J. (2021). Transcription regulates ribosome hibernation. Mol. Microbiol. 116, 663–673. doi: 10.1111/mmi.14762, PMID: 34152658 PMC8628635

[ref28] Feaga H. A. Kopylov M. Kim J. K. Jovanovic M. Dworkin J. (2020). Ribosome dimerization protects the small subunit. J. Bacteriol. 202:e00009-20. doi: 10.1128/JB.00009-20, PMID: 32123037 PMC7186458

[ref29] Gregory S. T. Carr J. F. Rodriguez-Correa D. Dahlberg A. E. (2005). Mutational analysis of 16S and 23S rRNA genes of *Thermus thermophilus*. J. Bacteriol. 187, 4804–4812. doi: 10.1128/JB.187.14.4804-4812.2005, PMID: 15995195 PMC1169515

[ref30] Helena-Bueno K. Chan L. Melnikov S. (2024a). Rippling life on a dormant planet: hibernation of ribosomes, RNA polymerases and other essential enzymes. Front. Microbiol. 15:1386179. doi: 10.3389/fmicb.2024.1386179, PMID: 38770025 PMC11102965

[ref31] Helena-Bueno K. Rybak M. Y. Ekemezie C. L. Sullivan R. Brown C. R. Dingwall C.. (2024b). A new family of bacterial ribosome hibernation factors. Nature 626, 1125–1132. doi: 10.1038/s41586-024-07041-8, PMID: 38355796 PMC10901736

[ref32] Hobbie S. N. Pfister P. Bruell C. Sander P. François B. Westhof E.. (2006). Binding of neomycin-class aminoglycoside antibiotics to mutant ribosomes with alterations in the a site of 16S rRNA. Antimicrob. Agents Chemother. 50, 1489–1496. doi: 10.1128/AAC.50.4.1489-1496.2006, PMID: 16569869 PMC1426975

[ref33] Hopes T. Norris K. Agapiou M. McCarthy C. G. P. Lewis P. A. O’Connell M. J.. (2022). Ribosome heterogeneity in *Drosophila melanogaster* gonads through paralog-switching. Nucleic Acids Res. 50, 2240–2257. doi: 10.1093/nar/gkab606, PMID: 34283226 PMC8887423

[ref34] Jenner L. Starosta A. L. Terry D. S. Mikolajka A. Filonava L. Yusupov M.. (2013). Structural basis for potent inhibitory activity of the antibiotic tigecycline during protein synthesis. Proc. Natl. Acad. Sci. 110, 3812–3816. doi: 10.1073/pnas.1216691110, PMID: 23431179 PMC3593886

[ref35] Kalapala S. K. Hobbie S. N. Böttger E. C. Shcherbakov D. (2010). Mutation K42R in ribosomal protein S12 does not affect susceptibility of *Mycobacterium smegmatis* 16S rRNA A-site mutants to 2-deoxystreptamines. PLoS One 5:e11960. doi: 10.1371/journal.pone.0011960, PMID: 20700526 PMC2916820

[ref36] Kessel R. G. (1966). An electron microscope study of nuclear-cytoplasmic exchange in oocytes of *Ciona intestinalis*. J. Ultrastruct. Res. 15, 181–196. doi: 10.1016/S0022-5320(66)80103-X, PMID: 5949056

[ref37] Killeavy E. E. Jogl G. Gregory S. T. (2020). Tiamulin-resistant mutants of the thermophilic bacterium *Thermus thermophilus*. Antibiotics 9:313. doi: 10.3390/antibiotics9060313, PMID: 32526926 PMC7345174

[ref38] Lang M. Krin E. Korlowski C. Sismeiro O. Varet H. Coppée J.-Y.. (2021). Sleeping ribosomes: bacterial signaling triggers RaiA mediated persistence to aminoglycosides. iScience 24:103128. doi: 10.1016/j.isci.2021.103128 34611612 PMC8476650

[ref39] Laurberg M. Kristensen O. Martemyanov K. Gudkov A. T. Nagaev I. Hughes D.. (2000). Structure of a mutant EF-G reveals domain III and possibly the fusidic acid binding site. J. Mol. Biol. 303, 593–603. doi: 10.1006/jmbi.2000.4168, PMID: 11054294

[ref40] Leesch F. Lorenzo-Orts L. Pribitzer C. Grishkovskaya I. Roehsner J. Chugunova A.. (2023). A molecular network of conserved factors keeps ribosomes dormant in the egg. Nature 613, 712–720. doi: 10.1038/s41586-022-05623-y, PMID: 36653451 PMC7614339

[ref41] Li Y. Sharma M. R. Koripella R. K. Banavali N. K. Agrawal R. K. Ojha A. K. (2021). Ribosome hibernation: a new molecular framework for targeting nonreplicating persisters of mycobacteria. Microbiology 167:1035. doi: 10.1099/mic.0.001035, PMID: 33555244 PMC8131030

[ref42] Li Y. Sharma M. R. Koripella R. K. Yang Y. Kaushal P. S. Lin Q.. (2018). Zinc depletion induces ribosome hibernation in mycobacteria. Proc. Natl. Acad. Sci. 115, 8191–8196. doi: 10.1073/pnas.1804555115, PMID: 30038002 PMC6094098

[ref43] Lin P. L. Flynn J. L. (2010). Understanding latent tuberculosis: a moving target. J. Immunol. 185, 15–22. doi: 10.4049/jimmunol.0903856, PMID: 20562268 PMC3311959

[ref44] Lin J. Zhou D. Steitz T. A. Polikanov Y. S. Gagnon M. G. (2018). Ribosome-targeting antibiotics: modes of action, mechanisms of resistance, and implications for drug design. Annu. Rev. Biochem. 87, 451–478. doi: 10.1146/annurev-biochem-062917-011942, PMID: 29570352 PMC9176271

[ref45] Lipońska A. Yap M.-N. F. (2021). Hibernation-promoting factor sequesters *Staphylococcus aureus* ribosomes to antagonize RNase R-mediated nucleolytic degradation. mBio 12:e0033421. doi: 10.1128/mBio.00334-21, PMID: 34253058 PMC8406268

[ref46] Mankin A. S. (1997). Pactamycin resistance mutations in functional sites of 16S rRNA. J. Mol. Biol. 274, 8–15. doi: 10.1006/jmbi.1997.1387, PMID: 9398510

[ref47] McKay S. L. Portnoy D. A. (2015). Ribosome hibernation facilitates tolerance of stationary-phase bacteria to aminoglycosides. Antimicrob. Agents Chemother. 59, 6992–6999. doi: 10.1128/AAC.01532-15, PMID: 26324267 PMC4604360

[ref48] McLaren M. Conners R. Isupov M. N. Gil-Díez P. Gambelli L. Gold V. A. M.. (2023). CryoEM reveals that ribosomes in microsporidian spores are locked in a dimeric hibernating state. Nat. Microbiol. 8, 1834–1845. doi: 10.1038/s41564-023-01469-w, PMID: 37709902 PMC10522483

[ref49] Nicholson D. Salamina M. Panek J. Helena-Bueno K. Brown C. R. Hirt R. P.. (2022). Adaptation to genome decay in the structure of the smallest eukaryotic ribosome. Nat. Commun. 13:591. doi: 10.1038/s41467-022-28281-0, PMID: 35105900 PMC8807834

[ref50] Noeske J. Huang J. Olivier N. B. Giacobbe R. A. Zambrowski M. Cate J. H. (2014). Synergy of streptogramin antibiotics occurs independently of their effects on translation. Antimicrob. Agents Chemother. 58, 5269–5279. doi: 10.1128/AAC.03389-14, PMID: 24957822 PMC4135883

[ref51] Osterman I. A. Wieland M. Maviza T. P. Lashkevich K. A. Lukianov D. A. Komarova E. S.. (2020). Tetracenomycin X inhibits translation by binding within the ribosomal exit tunnel. Nat. Chem. Biol. 16, 1071–1077. doi: 10.1038/s41589-020-0578-x 32601485

[ref52] Paternoga H. Crowe-McAuliffe C. Bock L. V. Koller T. O. Morici M. Beckert B.. (2023). Structural conservation of antibiotic interaction with ribosomes. Nat. Struct. Mol. Biol. 30, 1380–1392. doi: 10.1038/s41594-023-01047-y, PMID: 37550453 PMC10497419

[ref53] Pfister P. Hobbie S. Brüll C. Corti N. Vasella A. Westhof E.. (2005). Mutagenesis of 16S rRNA C1409-G1491 base-pair differentiates between 6′ OH and 6′ NH^3+^ aminoglycosides. J. Mol. Biol. 346, 467–475. doi: 10.1016/j.jmb.2004.11.073, PMID: 15670597

[ref54] Pfister P. Risch M. Brodersen D. E. Bottger E. (2003). Role of 16S rRNA helix 44 in ribosomal resistance to hygromycin B. Antimicrob. Agents Chemother. 47, 1496–1502. doi: 10.1128/AAC.47.5.1496-1502.2003, PMID: 12709313 PMC153343

[ref55] Polikanov Y. S. Blaha G. M. Steitz T. A. (2012). How hibernation factors RMF, HPF, and YfiA turn off protein synthesis. Science 336, 915–918. doi: 10.1126/science.1218538, PMID: 22605777 PMC3377384

[ref56] Polikanov Y. S. Szal T. Jiang F. Gupta P. Matsuda R. Shiozuka M.. (2014). Negamycin interferes with decoding and translocation by simultaneous interaction with rRNA and tRNA. Mol. Cell 56, 541–550. doi: 10.1016/j.molcel.2014.09.021, PMID: 25306922 PMC4334386

[ref57] Prezioso S. M. Brown N. E. Goldberg J. B. (2017). Elfamycins: inhibitors of elongation factor-Tu. Mol. Microbiol. 106, 22–34. doi: 10.1111/mmi.13750, PMID: 28710887 PMC5701666

[ref58] Prossliner T. Gerdes K. Sørensen M. A. Winther K. S. (2021). Hibernation factors directly block ribonucleases from entering the ribosome in response to starvation. Nucleic Acids Res. 49, 2226–2239. doi: 10.1093/nar/gkab017, PMID: 33503254 PMC7913689

[ref59] Rittershaus E. S. Baek S.-H. Sassetti C. M. (2013). The normalcy of dormancy: common themes in microbial quiescence. Cell Host Microbe 13, 643–651. doi: 10.1016/j.chom.2013.05.012, PMID: 23768489 PMC3743100

[ref60] Schlünzen F. Zarivach R. Harms J. Bashan A. Tocilj A. Albrecht R.. (2001). Structural basis for the interaction of antibiotics with the peptidyl transferase Centre in eubacteria. Nature 413, 814–821. doi: 10.1038/35101544, PMID: 11677599

[ref61] Schuwirth B. S. Day J. M. Hau C. W. Janssen G. R. Dahlberg A. E. Cate J. H. D.. (2006). Structural analysis of kasugamycin inhibition of translation. Nat. Struct. Mol. Biol. 13, 879–886. doi: 10.1038/nsmb1150, PMID: 16998486 PMC2636691

[ref62] Seely S. M. Parajuli N. P. De Tarafder A. Ge X. Sanyal S. Gagnon M. G. (2023). Molecular basis of the pleiotropic effects by the antibiotic amikacin on the ribosome. Nat. Commun. 14:4666. doi: 10.1038/s41467-023-40416-5, PMID: 37537169 PMC10400623

[ref63] Smith P. R. Pandit S. C. Loerch S. Campbell Z. T. (2022). The space between notes: emerging roles for translationally silent ribosomes. Trends Biochem. Sci. 47, 477–491. doi: 10.1016/j.tibs.2022.02.003, PMID: 35246374 PMC9106873

[ref64] Song S. Wood T. K. (2020). ppGpp ribosome dimerization model for bacterial persister formation and resuscitation. Biochem. Biophys. Res. Commun. 523, 281–286. doi: 10.1016/j.bbrc.2020.01.102, PMID: 32007277

[ref65] Stanley R. E. Blaha G. Grodzicki R. L. Strickler M. D. Steitz T. A. (2010). The structures of the anti-tuberculosis antibiotics viomycin and capreomycin bound to the 70S ribosome. Nat. Struct. Mol. Biol. 17, 289–293. doi: 10.1038/nsmb.1755, PMID: 20154709 PMC2917106

[ref66] Svidritskiy E. Ling C. Ermolenko D. N. Korostelev A. A. (2013). Blasticidin S inhibits translation by trapping deformed tRNA on the ribosome. Proc. Natl. Acad. Sci. 110, 12283–12288. doi: 10.1073/pnas.1304922110, PMID: 23824292 PMC3725078

[ref67] Thompson J. Cundliffe E. Dahlberg A. E. (1988). Site-directed mutagenesis of *Escherichia coli* 23S ribosomal RNA at position 1067 within the GTP hydrolysis Centre. J. Mol. Biol. 203, 457–465. doi: 10.1016/0022-2836(88)90012-5, PMID: 2462056

[ref68] Trauner A. Lougheed K. E. Bennett M. H. Hingley-Wilson S. M. Williams H. D. (2012). The dormancy regulator DosR controls ribosome stability in hypoxic mycobacteria. J. Biol. Chem. 287, 24053–24063. doi: 10.1074/jbc.M112.364851, PMID: 22544737 PMC3390679

[ref69] Trusiak M. Plitta-Michalak B. P. Michalak M. (2022). Choosing the right path for the successful storage of seeds. Plan. Theory 12:72. doi: 10.3390/plants12010072, PMID: 36616200 PMC9823941

[ref70] Tu D. Blaha G. Moore P. B. Steitz T. A. (2005). Structures of MLSBK antibiotics bound to mutated large ribosomal subunits provide a structural explanation for resistance. Cell 121, 257–270. doi: 10.1016/j.cell.2005.02.005 15851032

[ref71] Vila-Sanjurjo A. Schuwirth B.-S. Hau C. W. Cate J. H. (2004). Structural basis for the control of translation initiation during stress. Nat. Struct. Mol. Biol. 11, 1054–1059. doi: 10.1038/nsmb850, PMID: 15502846

[ref72] Wada A. Igarashi K. Yoshimura S. Aimoto S. Ishihama A. (1995). Ribosome modulation factor: stationary growth phase-specific inhibitor of ribosome functions from *Escherichia coli*. Biochem. Biophys. Res. Commun. 214, 410–417. doi: 10.1006/bbrc.1995.2302, PMID: 7677746

[ref73] Wada A. Yamazaki Y. Fujita N. Ishihama A. (1990). Structure and probable genetic location of a" ribosome modulation factor" associated with 100S ribosomes in stationary-phase *Escherichia coli* cells. Proc. Natl. Acad. Sci. 87, 2657–2661. doi: 10.1073/pnas.87.7.2657, PMID: 2181444 PMC53749

[ref74] Walter J. D. Hunter M. Cobb M. Traeger G. Spiegel P. C. (2012). Thiostrepton inhibits stable 70S ribosome binding and ribosome-dependent GTPase activation of elongation factor G and elongation factor 4. Nucleic Acids Res. 40, 360–370. doi: 10.1093/nar/gkr623, PMID: 21908407 PMC3245911

[ref75] Wang Y. J. Vaidyanathan P. P. Rojas-Duran M. F. Udeshi N. D. Bartoli K. M. Carr S. A.. (2018). Lso2 is a conserved ribosome-bound protein required for translational recovery in yeast. PLoS Biol. 16:e2005903. doi: 10.1371/journal.pbio.2005903, PMID: 30208026 PMC6135351

[ref76] Wells J. N. Buschauer R. Mackens-Kiani T. Best K. Kratzat H. Berninghausen O.. (2020). Structure and function of yeast Lso2 and human CCDC124 bound to hibernating ribosomes. PLoS Biol. 18:e3000780. doi: 10.1371/journal.pbio.3000780, PMID: 32687489 PMC7392345

[ref77] Wilson D. N. (2009). The A–Z of bacterial translation inhibitors. Crit. Rev. Biochem. Mol. Biol. 44, 393–433. doi: 10.3109/10409230903307311, PMID: 19929179

[ref78] Wilson D. N. (2014). Ribosome-targeting antibiotics and mechanisms of bacterial resistance. Nat. Rev. Microbiol. 12, 35–48. doi: 10.1038/nrmicro3155 24336183

[ref79] Wilson D. N. Schluenzen F. Harms J. M. Starosta A. L. Connell S. R. Fucini P. (2008). The oxazolidinone antibiotics perturb the ribosomal peptidyl-transferase center and effect tRNA positioning. Proc. Natl. Acad. Sci. 105, 13339–13344. doi: 10.1073/pnas.0804276105, PMID: 18757750 PMC2533191

[ref80] Xu W. Pagel F. T. Murgola E. J. (2002). Mutations in the GTPase center of *Escherichia coli* 23S rRNA indicate release factor 2-interactive sites. J. Bacteriol. 184, 1200–1203. doi: 10.1128/jb.184.4.1200-1203.2002 11807083 PMC134791

[ref81] Yoshida H. Maki Y. Furuike S. Sakai A. Ueta M. Wada A. (2012). YqjD is an inner membrane protein associated with stationary-phase ribosomes in *Escherichia coli*. J. Bacteriol. 194, 4178–4183. doi: 10.1128/JB.00396-12, PMID: 22661687 PMC3416271

